# Statins Improve Long Term Patency of Arteriovenous Fistula for Hemodialysis

**DOI:** 10.1038/srep22197

**Published:** 2016-02-23

**Authors:** Hao-Hsiang Chang, Yu-Kang Chang, Chia-Wen Lu, Chi-Ting Huang, Chiang-Ting Chien, Kuan-Yu Hung, Kuo-Chin Huang, Chih-Cheng Hsu

**Affiliations:** 1 Department of Family Medicine, National Taiwan University Hospital, Taipei, Taiwan; 2 Department of Life Science, National Taiwan Normal University, Taipei, Taiwan; 3 Institute of Population Health Sciences, National Health Research Institutes, Zhunan, Miaoli County, Taiwan; 4 Department of Medicine, National Taiwan University Hospital, Hsinchu branch, Hsinchu, Taiwan; 5 Department of Family Medicine, College of Medicine, National Taiwan University, Taipei, Taiwan; 6 Department of Health Services Administration, China Medical University, Taichung City, Taiwan; 7 Institute of Clinical Medicine, National Yang Ming University, Taipei, Taiwan

## Abstract

The protective effects of statins against stenosis for permanent hemodialysis access have been repeatedly demonstrated in animal studies, but remain controversial in human studies. This study aims to evaluate the association between statin use and permanent hemodialysis access patency using a nationwide hemodialysis cohort. A total of 9862 pairs of statin users and non-users, matched by age and gender, were selected for investigation from 75404 new hemodialysis patients during 2000–2008. The effect of statins on permanent hemodialysis access patency was evaluated using Cox proportional hazards models. Compared with non-users, statin users had an overall 18% risk reduction in the composite endpoint in which angioplasty and recreation were combined (adjusted hazard ratio = 0.82 [95%CI, 0.78–0.87]) and 21% in recreation of permanent hemodialysis access (adjusted hazard ratio = 0.79 [95%CI, 0.69–0.80]). Specifically, the protective effect was found for arteriovenous fistula (adjusted hazard ratio = 0.78[95% CI, 0.73–0.82] for composite endpoint and 0.74 [95% CI, 0.69–0.80] for vascular recreation), but not for arteriovenous grafts (adjusted hazard ratio = 1.10 [95% CI, 0.98–1.24] and 0.94 [95% CI, 0.83–1.07]). Statins possess a protective effect for arteriovenous fistula against the recreation of permanent hemodialysis access. The results provide a pharmaco-epidemiologic link between basic research and clinical evidence.

Autogenous arteriovenous fistula (AVF) is the universally recommended permanent hemodialysis (HD) access for patients receiving hemodialysis^1^,^2^,^3^. The maintenance of AVF patency remains a challenge for current medicine. Several advances in medical technologies have been made that help maintain AVF patency. These include ultrasound assessment for operation, better timing for the first cannulation, advances in cannulation techniques, and far infrared therapy^4^,^5^,^6^. Even with these approaches detailed in guidelines for creation and care, AVF stenosis rates are still far from optimal. According to a recent systematic review, 1-year patency rates are 62~68%, and 2-year patency rates are 38~56%^7^.

To overcome this difficulty, the physiological mechanisms of AVF stenosis are widely studied and proposed to be affected mainly by intimal hyperplasia and inappropriate outward remodeling^8^. On the basis of these understandings, some medications possessing potentially beneficial effects on AVF patency have been tested in clinical studies^9^,^10^,^11^, such as angiotensin converting enzyme inhibitors (ACEIs), angiotensin II receptor blockers (ARBs), anti-platelets, and anti-coagulants. However, no medication has been consistently reported to possess beneficial effects for AVF patency. Among the candidate medicines for alleviating AVF stenosis, statins have received special scrutiny. Statins are well known to reduce inflammation and improve endothelial function beyond lowering cholesterol in end stage renal disease patients. In animal studies, statins have been demonstrated to improve blood flow, endothelial function and prevent stenosis of AVF^12^,^13^,^14^. Though statins possess these potential protective effects on AVF patency in basic research, several clinical studies have failed to show any association between improved survival of permanent HD access and the prescription of statins^9^,^15^,^16^,^17^. Only one small-sample study has demonstrated a beneficial effect^18^, meaning that statins have never been proven to reduce AVF stenosis in a large study of human dialysis patients. This study aims to evaluate the effect of statins on the AVF long term patency through a nation-wide analysis cohort.

## Results

### Study subjects’ characteristics

The demographic characteristics, comorbid diseases and medication exposures of statin users and non-users are listed on Table 1. The mean age of statin users was 60.1 ± 14.8 years, and 57.7% were women. The matched non-users had almost identical mean age and gender percentages. A significant difference found between statin users and non-users is that a higher percentage of the control group lived in rural areas. Compared with statin non-users, the statin users were more likely to be co-morbid with congestive heart failure, cerebral vascular accident, peripheral vascular disease, diabetes and atherosclerotic heart disease. However, they were less likely to have had liver disease and cancers. Statin users were also more likely to be treated with aspirin, clopidogrel, warfarin, anti-diabetic medicine, and all categories of antihypertensives.

### Association between statin use and permanent HD access patency

**Figure 1:**
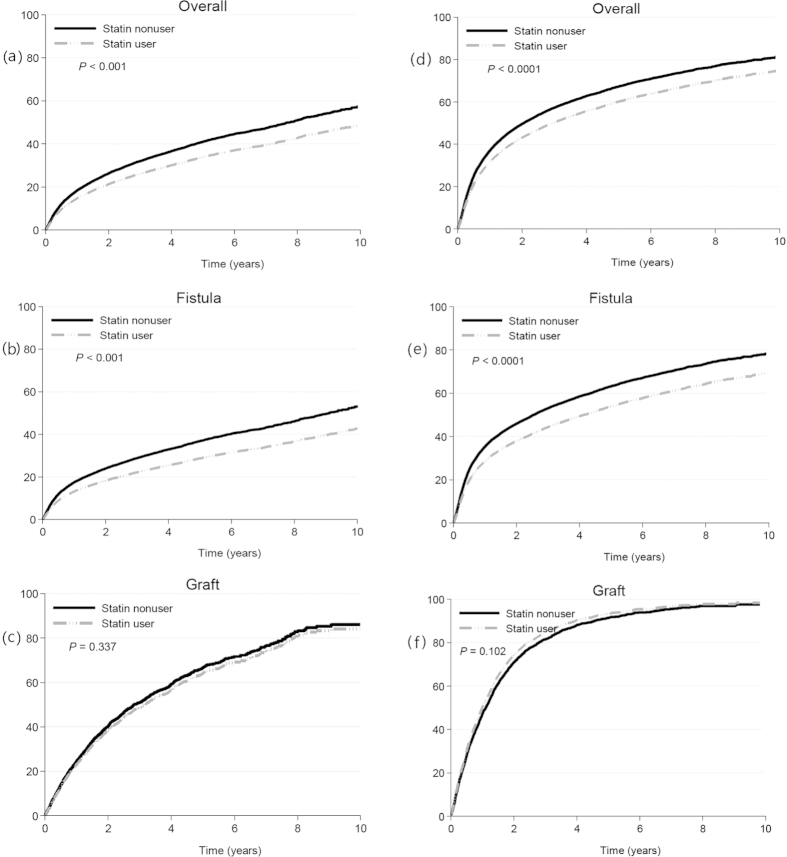
Cumulative incidence of permanent hemodialysis access recreation and the composite endpoint for statin users and non-users by the multivariate Nelson-Aalen method. Statin users had significantly lower incidence rates of permanent hemodialysis access recreation than statin user group in (**a**) overall pairs and (**b**) arteriovneous fistula pairs, but not in (**c**) arteriovenous graft pairs. Incidence rates of composite endpoint, permanent hemodialysis access recreation or angioplasty, for statin users were significantly lower than nonusers in (**d**) overall pairs (**e**) and arteriovneous fistula pairs (**f**), but not in arteriovenous graft pairs.

After a mean follow-up period of 3.34 years in statin user group and 3.79 years in non-user group, the incidence rates of permanent HD access recreation were 9.83 and 10.23 per 100 person-years, respectively, resulting in a crude hazard ratio of 0.98 (95% CI, 0.93 to 1.03) and an adjusted hazard ratio of 0.79 (95% CI, 0.74 to 0.84; P < 0.001). The effect of statins on permanent HD access recreation was calculated using Cox regression model and is demonstrated on Fig. 1. As for the AVFs, the crude and adjusted hazard ratios of permanent HD access recreation were 0.94 (95% CI, 0.89 to 0.99; P < 0.05) and 0.74 (95% CI, 0.69 to 0.80; P < 0.001). Meanwhile, for the patients with arteriovenous graft (AVG), the crude and adjusted hazard ratios were 0.99 (95% CI, 0.90 to 1.09) and 0.94 (95% CI, 0.83 to 1.07). We also performed survival analysis using a composite endpoint consisting of angioplasty and permanent HD access recreation to test the effect of statin use on the patency of permanent HD access. The adjusted hazard ratios of the composite endpoint were 0.78 (95%CI; 0.73–0.82) for AVF, 1.10 (95% CI; 0.98–1.24) for AVG and 0.82 (95% CI; 0.78–0.87) across both types of permanent HD access. The hazard ratios of permanent HD access recreation and the composite endpoint for the statin users compared to the non-users are listed on Table 2.

To further verify if statins’ protective effect matched the dose-response relation, prescription days were used as a dose indicator; statin users were then stratified into quartiles by prescription days. The adjusted hazard ratios for permanent HD access recreation in the AVF patients from Q1 to Q4 were 0.82 (95% CI, 0.75 to 0.90), 0.72 (95% CI, 0.65 to 0.80), 0.72 (95% CI, 0.65 to 0.80), and 0.67 (95% CI, 0.60 to 0.75). In AVG patients, the hazard ratios were 1.02 (95% CI, 0.86 to 1.21), 1.13 (95% CI, 0.96 to 1.33), 0.85 (95% CI, 0.72 to 1.01), and 0.74 (95% CI, 0.62 to 0.89).

### Other factors associated with permanent HD access recreation

Using a multivariate survival model, the hazard ratios of other factors including age, sex, comorbid diseases, and other medicines prescribed before the index date are listed on Table 3. Older age, female, peripheral vascular disease and gastrointestinal diseases were shown to increased risk for permanent HD access recreation in the AVF patients. Of all the medicines covered in this study, ARBs were associated with a reduced risk (HR, 0.92, 95%CI, 0.87 to 0.98; P < 0.05); however, loop diuretics, thiazides, alpha glucosidase inhibitors and digoxin were associated with increased risks of permanent HD access recreation in AVF patients. In AVG patients, thiazides and insulin were associated with increased risks of permanent HD recreation.

### The protective effect on permanent HD access recreation among various statins

To determine whether the protective effects on permanent HD access recreation differ across the various statins, a subgroup analysis was performed. Four out of six statins showed significant protective effects on permanent HD access, but lovastatin 0.90 (95% CI, 0.71 to 1.14) and fluvastatin 0.84 (95% CI, 0.70 to 1.02) failed to demonstrate statistically significant protective effects. The crude and adjusted hazard ratios for permanent HD access recreation across different statins are shown on Table 4.

### Propensity score matched cohort, time dependent analysis and competing risk survival analysis

To minimize potential bias in the differences between statin users and non-users, we created a propensity score matched cohort, containing 3,864 matched pairs. The baseline characteristics of the cohort can be found online as supplementary Table S1. The R square of logistic regression model used for creating propensity scores is 0.4254 by Cox & Snell R-square or 0.6254 by Nagelkerke R-square (see supplementary Table S2). The cohort was then used for the following 3 analyses to test the protective effect of statins on permanent HD access. A Cox regression model was used to calculate the statin-users’ hazard ratios for permanent HD access recreation. As shown in Table 5, the hazard ratios for statin-users were 0.88 (95%CI; 0.80–0.97) for AVF, 1.09 (95% CI; 0.93–1.29) for AVG, and 0.94 (95% CI; 0.86–1.01) for across both types. For further clarification on the association of statin use and permanent HD access recreation, a time-dependent Cox analysis was performed and the results are provided online in supplementary Table S3. The hazard ratios for permanent HD access recreation for statin-users were 0.80 (95%CI; 0.72–0.90) for AVF, 0.84 (95% CI; 0.70–1.00) for AVG and 0.80 (95% CI; 0.73–0.88) for across both types. Lastly, competing risks adjusted survival analysis was done and the results are shown online in supplementary Table S4. Death and renal transplantation were taken as the competing risks. The adjusted hazard ratios for permanent HD access recreation for statin-users were 0.87 (95%CI; 0.79–0.96) for AVF, 1.07 (95% CI; 0.90–1.25) for AVG and 0.92 (95% CI; 0.85–1.00) for across both types.

## Discussions

The present study demonstrates that statins are associated with an 18% risk reduction in the composite endpoint that incorporated angioplasty and permanent HD access recreation and 21% in recreation of permanent HD access. The risk reduction effect of statins was found in the autogenous AVF group, but not generally in the AVG group. The protective effect in AVF was found to respond to a dose-index, surrogated by days of prescription. A significant protective effect was found in the AVG patients with the highest range of prescription days. It is reasonable to assume that statins could also exert a milder beneficial effect in AVG patients than in AVF patients. The finding of such a beneficial effect of statins is the first ever reported on the basis of a large scale nation-wide population study.

The randomized controlled clinical trial is the gold standard for confirming the efficacy of an intervention. However, a large observational study, such as ours, provides a unique opportunity to study possible effects of a pharmacological intervention, often without the sample size and ethical limitations of a clinical trial. The potential confounding factors are usually problematic and should be collected and adjusted in such an analysis. In the present study, we further recruited a propensity score matched cohort and re-calculated the hazard ratios by multivariate Cox regression to minimize the potential bias resulting from baseline differences. The results indicate that statins prevent AVF from permanent HD access recreation. Mortality and renal transplantation cases were censored in our survival analysis of permanent HD access, which could result in potential bias from these competing risks^19^. The results of competing risk analysis also approve the protective effect of statins on AVF. Furthermore, the statin users are more frequently co-morbid with peripheral vascular disease and diabetes, which may increase risk of AVF failure. The protective effect is thus potentially underestimated in this non-intentional study. Taken these findings together, we conclude that statin users had a lower permanent HD access recreation hazard for AVF.

Statins have been repeatedly shown to possess beneficial effects on the prevention of AVF stenosis in animal and cellular studies. Simvastatin had been shown to reduce venous neo-intimal hyperplasia and vascular smooth muscle proliferation by decreasing the expression of vascular endothelial growth factor A (VEGF-A) and matrix metalloproteinase 9 (MMP-9)^14^. Rosuvastatin had been demonstrated to increase the blood flow in the venous limb of AVF in diabetic rats, which was associated with an anti-inflammatory effect and resulting from endothelial function improvement^13^. Atovastatin has been shown to decrease proliferation, migration, and the passage of human smooth muscle cells (HSMC) across a matrix barrier^20^. Pravastatin was reported to reduce intimal hyperplasia in mice that was associated with decreased vascular smooth muscle cell (VSMC) proliferation and platelet-derived growth factor-induced VSMC migration and inhibited macrophage migration^21^. Statins improve long term AVF patency through their potential ability of endothelial function improvement and inhibition of vascular smooth muscle proliferation and migration.

Another concern of permanent HD access failure is the vascular calcification which is commonly seen in hemodialysis patients. The severity of vascular calcifications has been shown to be associated with AVF failure^22^. As well as this, diabetes and male gender have been identified as risk factors for vascular calcification^22^. Though the data of vascular calcification are not available in the present study, more diabetic patients were in the statin-user group, the patients with statins thus theoretically had more pronounced vascular calcification than non-users. This condition should hinder the protective effect of statin on AVF and make the findings in the current study more conservative. In the analysis of the propensity score matched subcohort, in which the prevalent rate of diabetes in statin users and non-users are similar, the protective effect of statins still exists. Taken these vascular calcification related conditions into consideration, the present study demonstrates the beneficial effect of statins on AVF failure.

Several previous studies have debated the relationship between statins and outcomes of permanent HD access for hemodialysis. Righette *et al.* showed that statins possess a beneficial effect on AVF survival but it was not clear about the generalizability of this small-scale study which was conducted in a single center^18^. Saran *et al.* evaluated the association between specific medicines and AVF outcomes^9^. The study concluded that statins are not associated with better permanent HD access outcomes based on Dialysis Outcomes and Practice Patterns Study (DOPPS), but the study was confined to United States patients. Another study by Andreucci *et al.* based on DOPPS using US, European and Japanese data found the ineffectiveness of statins on permanent HD access patency^23^. Yevzlin *et al.* evaluated the relationship between medication use and permanent HD access patency using the cohort of US Renal Data System Dialysis Mortality and Morbidity Wave II study, and also reported that statins were not associated with better permanent HD access patency^11^. These hemodialysis cohort studies had a similar design to the present study but failed to demonstrate the beneficial effect of statins. Apart from the ethnical and sampling differences, the critical difference between the current study and others is the definition of statin users. To further clarify the association between statin use and permanent HD access recreation, statin use was defined as a time dependent variable. The hazard ratios generated from the time-dependent analysis demonstrate that statin use is a protective factor of permanent HD access recreation for AVF. Pisoni *et al.*^16^ and Birch *et al.*^15^ both based on restricted results from a single-center study, reported that statin users were not associated with improved permanent HD access patency. Florescu and Birch stated that single statin use may not be effective enough to treat the complex pathology of AVF stenosis^17^. Whether the statin use before permanent HD access creation decreases risk of permanent HD accessrecreation remains unclear. Current guidelines do not recommend the routine statin use in ESRD patients. The present study demonstrates that the statin use after permanent HD access creation possess a significant risk reduction effect on permanent HD access recreation by propensity score matched, time-dependent and competing risk survival analyses.

Four out of six statins investigated in the current study demonstrated a significant risk reduction effect on the AVF failure rate. Meanwhile, lovastatin and fluvastatin showed a risk lowering trend but not one that was statistically significant. Both of them are categorized as moderate to low potency^24^. This finding suggests that the beneficial effect on permanent HD access outcomes is a universal but that the effect depends on the statin’s potency categorization. The differential effects among individual statins should be clarified in future studies.

Older age and being female are demographic features associated with increased permanent HD access recreation for AVF in this study. Older age was uniformly recognized as one risk factor of unassisted and assisted permanent HD access failure in the previous studies^4^,^16^,^25^,^26^,^27^. Female patients were shown to have higher AVF failure rates in several recent studies^28^, but not in other studies^4^,^9^,^15^,^16^. One meta-analysis reported that female gender was not a significant risk factor of permanent HD access failure^29^. We found that peripheral vascular disease(PVD) was associated with increased permanent HD access recreation, which is compatible with previous studies^4^,^26^,^27^,^28^. However, congestive heart failure, cerebral vascular disease and diabetes were not identified as significant risk factors of AVF or AVG failure in this analysis. PVD is thought to be the indicator of late cardiovascular diseases. These results may indicate that not only the comorbid conditions but also the severity of cardiovascular disease contribute to permanent HD access stenosis. Future application of statins for AVF protection should take age, gender and the severity of cardiovascular disease into consideration.

Regarding permanent HD access protection, angiotensin converting enzyme inhibitors (ACEI) and angiotensin II receptor antagonists (ARB) are also drugs of choice for their pleiotrophic effects. Saran’s study based on DOPPS reported that ACEIs were associated with better assisted patency in AVFs, that calcium channel blockers (CCBs) were associated with better unassisted patency in AVGs and that aspirin was associated with better assisted patency in AVGs^9^. Our findings suggest a benefit of ARBs in AVF patients. No other antihypertensive agents including ACEI and CCB were found to be beneficial for reducing permanent HD access failure. In another retrospective study, ARB was also noted to be beneficial for AVF patency in ACE DD genotype patients^30^. Anti-platelets were reported by a meta-analysis to increase the short term patency of AVFs and AVGs^31^. In our analysis, neither aspirin nor clopidogrel was related to a decreased risk of permanent HD access recreation. To elucidate the relationships between these medications and permanent HD access outcomes requires further laboratory and clinical studies.

The proportion of incident hemodialysis patients in Taiwan during 2000–2008 who received autogenous AVF was 83.9%, which exceeds the goal of the AVF First Initiative. The incidence rate of permanent HD access recreation in the AVF group was much lower than that in the AVG group. The findings re-confirm the superiority of AVF to AVG from the viewpoint of permanent HD access failure. Concerned that AVF first is associated with higher primary failure and prolonged catheter dependence, some researchers debated whether the AVF first policy is sensible for all patients^32^,^33^. In general, AVF first was proved to be more cost-effective than AVG for long term hemodialysis^34^,^35^,^36^. For some subgroups such as the elderly, those with limited life expectancy, and pediatric patients, individual assessment of primary failure risk including patients’ preference should be done before permanent HD access creation.

This is a retrospective study using a national health insurance dataset. Several limitations may exist in this kind of analysis. First, when using administrative databases, the identification of comorbidities, vascular access creation type, medications are based on ICD-9 disease and procedure codes, so misclassification inevitably occurs. However, the misclassification is often non-differential and the outcome difference is toward null with a large sample. There were no enough information to define the permanent HD access failure was primary or secondary. Second, some specific individual information such as smoking, drinking and the location of the permanent HD access was not available through administrative data in the National Health Insurance Research Database (NHIRD). Third, we were also unable to obtain detailed laboratory data, such as serum creatinine, cholesterol level, C reactive protein, urinary protein excretion and the level of vascular calcification.

In summary, our findings suggest that statins use after permanent HD access creation possess a dose-responsive effect of protecting AVF from stenosis for patients undertaking hemodialysis. The beneficial effect on permanent HD access outcomes is a universal class effect and the effect size is associated with the statins’ potency. The use of statin may reduce failure of AVF, therefore, promoting patient outcomes and reducing health-care costs. These results have important therapeutic implications for future prospective randomized control studies.

## Methods

### Data source

The data was extracted from the Taiwanese NHIRD, which contains the healthcare utilization information of about 99% of the 23 million people enrolled in the universal National Health Insurance Program. The information kept in the NHIRD includes age, gender, residency area, income, diagnosis codes, and medications. We used the International Classification of Diseases, Ninth Revision (ICD-9) to define investigated comorbid diseases. The study was approved by the institutional review board at the National Health Research Institutes. The methods were carried out in accordance with the approved guidelines.

### Design and Study subjects

We used a population-based retrospective cohort study design to evaluate the relationship between statin use and recreation of permanent HD access in patients under hemodialysis. A regular hemodialysis cohort older than 20 years who received dialysis treatment for at least 3 months during 2000 to 2008 was selected in this study. From this cohort, we identified 40,459 study subjects who survived more than 2 years after starting hemodialysis, did not shift to peritoneal dialysis within 2 years after the dialysis commencement, and received their first permanent HD access operation 1 year prior to or after their first dialysis.

The status of statin exposure was scrutinized for 2 years after the permanent HD access creation. Those who had been prescribed statins for at least 30 days within 1 year were defined as statin users (n = 11,297). The first date of statin prescription was assigned as the index date. For those who took statins before the permanent HD access creation, the index date was assigned as the date of the permanent HD access creation. The statin users were then matched by age and gender to their nonuser counterparts (n = 29,161) in a 1:1 ratio. Finally, 9,826 statin user and nonuser pairs were identified for further analysis. Of the study subjects, 16,700 (85%) received autogenous arteriovenous fistula (AVF) and 2,952 (15%) received arteriovenous graft (AVG).The index date of the statin nonusers was assigned as the same index date for each individual pair. The study subjects were followed through until permanent HD access recreation (the primary outcome), kidney transplantation, death, or December 31, 2008, whichever came first.

### History of comorbidities and medications

From the NHIRD, we collected patients’ information about age, gender, residency area (city, township and rural area), income level (low, middle and high), and pre-dialysis comorbidities defined by the ICD-9 disease or procedure coding for at least 1 hospitalization or 2 ambulatory visits within 1 year due to some important chronic illnesses. The comorbidities investigated in this study included congestive heart failure (398.91, 422, 425, 428, 402.x1, 404.x1, 404.x3, V42.1), cerebrovascular disease (430–438), peripheral vascular disease (440–444, 447, 451–453, 557), diabetes (250, 357.2, 362.0x, 366.41), atherosclerotic heart disease (410–414, V45.81, V45.82), other heart disease (420–421, 423–424, 429, 785.0–785.3, V42.2, V43.3), chronic obstructive pulmonary disease (491–494, 496, 510), gastrointestinal disease (456.0–456.2, 530.7, 531–534, 569.84, 569.85, 578), liver disease (570, 571, 572.1, 572.4, 573.1–573.3, V42.7), dysrhythmia (426–427, V45.0, V53.3), cancer (140–172, 174–208, 230–231, 233), percutaneous coronary interventions (00.66, 36.01, 36.02, 36.05, 36.06, 36.07), and implantable cardioverter defibrillators/cardiac resynchronization therapy with defibrillator (37.94).

In addition to the statins, other pre-dialysis medications used were also controlled in this study, which included non-steroid anti-inflammatory drugs (NSAIDs), acetaminophen, aspirin, antihypertensive agents (angiotensin-converting enzyme inhibitor, angiotensin receptor blocker, alpha-blocker, beta-blocker, acetazolamide, calcium channel blocker and diuretics), anti-diabetic drugs (biguanide, sulfonylurea, alpha glucosidase inhibitor, thiazolidinedione, meglitinide, and insulin), morphine, warfarin, clopidogrel and digoxin.

### Statistical analysis

Baseline characteristics of the study subjects were described as the frequencies with percentages for categorical variables and means with standard deviation for continuous variables. We also used 1:1 propensity score matching^37^ to recruit the similar two groups with known confounders to balance the different baseline characteristics between statin users and nonusers that might confound the outcomes. The Cox proportional hazards model was used to estimate the hazard ratio (HR) and 95% confidence interval (CI) for the risk of permanent HD access recreation compared between the statin users and nonusers. The cumulative hazards of permanent HD access recreation over time were calculated using the Nelson-Aalen method^38^ to adjust the covariates adopted in the Cox proportional hazards models. We used log–log survival plots for all time-independent covariates to test the proportional hazard assumption and confirmed all assessed graphs did not violate the assumption. Study entry was defined as the index date. Observations were censored on December 31, 2008, the date patients died, or the date patients received kidney transplantation, whichever occurred first. The covariates adjusted in the multivariate Cox hazards models included age, gender, residency area, income level, and pre-dialysis medication use and comorbidities. During the follow-up period, because the number of death (698 [21.9%] for statin nonusers and 769 [19.9%] for statin users) and the number of those who received kidney transplant (108 [3.4%] for statin nonusers and 220 [5.7%] for statin users) could not be ignored, we further extended the Cox hazards models for competing risk adjustment, by considering the subdistribution hazard^39^.All p values were 2-sided, and the p value < 0.05 was considered to be a significance level. All analysis was conducted using the SAS version 9.3 (SAS Institute Inc, Cary, North Carolina).

## Additional Information

**How to cite this article**: Chang, H.-H. *et al.* Statins Improve Long Term Patency of Arteriovenous Fistula for Hemodialysis. *Sci. Rep.* **6**, 22197; doi: 10.1038/srep22197 (2016).

## Supplementary Material

Supplementary Information

## Figures and Tables

**Table 1:** Demographic characteristics, comorbid diseases and medications exposure between statin users and nonusers.

	Statin nonusers		Statin users		
	N = 9,826		N = 9,826		P value
Follow up time (year)					
Means (SD)	3.79	(2.76)	3.34	(2.34)	<0.0001
Age (year)					
Means (SD)	60.0	(11.6)	60.1	(11.5)	0.8989
Sex (n, %)					1.0000
Male	4157	(42.3)	4157	(42.3)	
Female	5669	(57.7)	5669	(57.7)	
Location (n, %)					<0.0001
City	2398	(24.4)	2779	(28.3)	
Township	3001	(30.5)	3328	(33.9)	
Rural area	4427	(45.1)	3719	(37.8)	
Comorbidity					
CHF	774	(7.9)	1061	(10.8)	<0.0001
CVA	1286	(13.1)	2152	(21.9)	<0.0001
PVD	629	(6.4)	985	(10.0)	<0.0001
DM	3941	(40.1)	7113	(72.4)	<0.0001
ASHD	2284	(23.2)	3593	(36.6)	<0.0001
Other heart disease	1155	(11.8)	1256	(12.8)	0.0281
COPD	1450	(14.8)	1436	(14.6)	0.7778
GI disease	2884	(29.4)	2874	(29.3)	0.8755
Liver disease	1424	(14.5)	1033	(10.5)	<0.0001
Cancer	695	(7.1)	549	(5.6)	<0.0001
Medication use					
NSAID	6970	(70.9)	7228	(73.6)	<0.0001
Aspirin	2676	(27.2)	4607	(46.9)	<0.0001
Clopidogrel	436	(4.4)	1335	(13.6)	<0.0001
Warfarin	217	(2.2)	264	(2.7)	0.0300
ACEI	3558	(36.2)	4244	(43.2)	<0.0001
ARB	3213	(32.7)	5189	(52.8)	<0.0001
Beta-blocker	4675	(47.6)	6123	(62.3)	<0.0001
Non-DHP CCB	1476	(15.0)	1904	(19.4)	<0.0001
DHP CCB	7110	(72.4)	8278	(84.3)	<0.0001
Biguanide	890	(9.1)	1920	(19.5)	<0.0001
Sulfonylurea	1918	(19.5)	3956	(40.3)	<0.0001
Alpah glucosidase inhibitor	373	(3.8)	1375	(14.0)	<0.0001
Thiazolidinedione	314	(3.2)	1299	(13.2)	<0.0001
Meglitinide	859	(8.7)	2320	(23.6)	<0.0001
Insulin	2572	(26.2)	4840	(49.3)	<0.0001
Statin	0	(0.0)	5075	(51.7)	<0.0001

Abbreviations: CHF, congestive heart failure; CVA, cerebral vascular accident; PVD, peripheral vascular disease; DM, diabetes mellitus; ASHD, atherosclerotic heart disease; COPD, chronic obstructive pulmonary disease; GI, gastrointestinal; NSAID, non steroid anti-inflammatory drug; ACEI, angiotensin converting enzyme inhibitor; ARB, angiotensin II receptor blocker; DHP, dihydropyridine; CCB, calcium channel blocker.

**Table 2:** Crude and adjusted hazard ratio of permanent hemodialysis access recreation and the composite endpoint for statin users and nonusers.

	N	Event	Person-years	Event Rate (per 100 person-years)	Hazard ratio	Adjusted hazard ratio
Vascular access recreation						
Overall						
Statin nonusers	9826	3665	37275.01	9.83	1.0	1.0
Statin users	9826	3355	32780.10	10.23	0.98 (0.93–1.03)	0.79 (0.74–0.84)**
AVF						
Statin nonusers	8519	2882	33823.15	8.52	1.0	1.0
Statin users	8181	2431	28727.13	8.46	0.94 (0.89–0.99)*	0.74 (0.69–0.80)**
AVG						
Statin nonusers	1307	783	3451.86	22.68	1.0	1.0
Statin users	1645	924	4052.97	22.80	0.99 (0.90–1.09)	0.94 (0.83–1.07)
Composite endpoint						
Overall						
Statin nonusers	8865	5389	25278.18	21.32	1.0	1.0
Statin users	8649	5361	20587.80	26.04	1.13 (1.08–1.17)**	0.82 (0.78–0.87)**
AVF						
Statin nonusers	7701	4405	23440.71	18.79	1.0	1.0
Statin users	7235	4115	18686.30	22.02	1.09 (1.04–1.14)**	0.78 (0.73–0.82)**
AVG						
Statin nonusers	1164	984	1837.48	53.55	1.0	1.0
Statin users	1414	1246	1901.51	65.53	1.18 (1.08–1.28)**	1.10 (0.98–1.24)

^*^p < 0.05; **p < 0.001

Composite endpoint consists of angioplasty and vascular access recreation

Covariates adjusted in multivariate models included income, age, sex, area, CHF, CVA, PVD, DM, ASHD, COPD, GI disease, liver disease, dysrhythmia, CABG, PCI, ICD, drugs prescribed before index date (included NSAID, aspirin, ACEI, ARB, beta-blocker, non-DHP and DHP CCB, biguanides, sulfonylureas, alpha glucosidase inhibitors, thiazolidinediones, meglitinides, insulin, DPP4 inhibitors, statins, warfarin, clopidogrel).

**Table 3:** Risk factors of permanent hemodialysis access recreation by multivariate Cox proportional hazards model by different types of permanent hemodialysis access.

	Overall		AVF		AVG	
	HR	95% CI	HR	95% CI	HR	95% CI
Statin	0.79	(0.74–0.84)**	0.74	(0.69–0.80)**	0.94	(0.83–1.07)
Age (ref = 20–39)						
40–49	1.03	(0.90–1.17)	0.99	(0.85–1.14)	1.20	(0.83–1.74)
50–59	1.14	(1.01–1.30)*	1.09	(0.95–1.25)	1.18	(0.84–1.67)
60–69	1.29	(1.14–1.46)**	1.20	(1.05–1.38)*	1.31	(0.93–1.85)
≧70	1.49	(1.31–1.70)**	1.36	(1.18–1.56)**	1.47	(1.04–2.08)*
Male	0.82	(0.78–0.86)**	0.86	(0.82–0.91)**	0.94	(0.84–1.05)
Comorbidity						
CHF	1.07	(0.98–1.15)	1.09	(0.99–1.20)	0.90	(0.78–1.06)
CVA	1.03	(0.97–1.10)	0.99	(0.92–1.07)	0.99	(0.88–1.11)
PVD	1.09	(1.01–1.18)*	1.13	(1.03–1.25)*	0.89	(0.76–1.04)
DM	1.06	(0.99–1.14)	1.06	(0.98–1.15)	1.00	(0.87–1.15)
ASHD	1.07	(1.01–1.13)*	1.06	(0.99–1.14)	1.10	(0.99–1.24)
COPD	1.05	(0.99–1.12)	1.03	(0.95–1.11)	1.09	(0.96–1.24)
GI disease	1.10	(1.04–1.15)**	1.11	(1.04–1.18)**	0.95	(0.86–1.05)
Liver disease	0.99	(0.92–1.06)	0.99	(0.91–1.07)	0.90	(0.78–1.04)
Cancer	1.12	(1.02–1.23)*	1.10	(0.98–1.23)	0.97	(0.82–1.14)
Drug						
NSAID	1.08	(1.02–1.14)*	1.04	(0.97–1.11)	1.12	(0.98–1.27)
Aspirin	1.05	(0.99–1.11)	1.04	(0.97–1.11)	0.97	(0.87–1.08)
Clopidogrel	0.94	(0.86–1.03)	0.90	(0.80–1.01)	1.03	(0.87–1.21)
Warfarin	1.18	(1.03–1.37)*	1.07	(0.89–1.30)	0.99	(0.79–1.24)
ACEI	0.96	(0.92–1.02)	0.99	(0.94–1.05)	0.91	(0.82–1.01)
ARB	0.93	(0.88–0.98)*	0.92	(0.87–0.98)*	0.96	(0.86–1.07)
Beta blocker	0.99	(0.94–1.05)	1.00	(0.95–1.07)	0.94	(0.84–1.04)
Non DHP CCB	0.96	(0.90–1.02)	0.96	(0.89–1.04)	1.00	(0.88–1.14)
DHP CCB	0.98	(0.92–1.05)	0.99	(0.92–1.07)	0.96	(0.83–1.10)
Biguanides	1.06	(0.98–1.15)	1.06	(0.97–1.16)	1.10	(0.95–1.29)
Sulfonylureas	1.02	(0.95–1.09)	1.03	(0.95–1.12)	1.04	(0.91–1.19)
AGI	1.10	(1.01–1.20)*	1.13	(1.01–1.25)*	1.00	(0.84–1.18)
TZD	0.96	(0.88–1.06)	0.91	(0.82–1.02)	1.13	(0.95–1.34)
Meglitinides	0.97	(0.90–1.04)	0.97	(0.89–1.06)	0.96	(0.84–1.10)
Insulin	1.09	(1.03–1.16)*	1.05	(0.98–1.13)	1.25	(1.11–1.41)**

^*^p < 0.05; **p < 0.001

Abbreviations: CHF, congestive heart failure; CVA, cerebral vascular accident; PVD, peripheral vascular disease; DM, diabetes mellitus; ASHD, atherosclerotic heart disease; COPD, chronic obstructive pulmonary disease; GI, gastrointestinal; NSAID, non steroid anti-inflammatory drug; ACEI, angiotensin converting enzyme inhibitor; ARB, angiotensin II receptor blocker; DHP, dihydropyridine; CCB, calcium channel blocker; AGI, alpha glucosidase inhibitor; TZD, thiazolidinediones.

**Table 4:** Crude and adjusted hazard ratio of permanent hemodialysis access recreation for different statins.

	N	event	Person-years	Incidence (%)	HR	Adjused HR
Atorvastatin						
Nonusers	4693	1739	17723.87	9.81	1.0	1.0
Users	4693	1612	15521.13	10.39	0.99 (0.92–1.06)	0.79 (0.72–0.87)**
Lovastatin						
Nonusers	621	214	2387.40	8.96	1.0	1.0
Users	621	232	2265.60	10.24	1.13 (0.94–1.36)	0.90 (0.71–1.14)
Simvastatin						
Nonusers	1616	602	6321.88	9.52	1.0	1.0
Users	1616	566	6050.86	9.35	0.96 (0.86–1.08)	0.72 (0.61–0.84)**
Rosuvastatin						
Nonusers	1115	415	4224.19	9.82	1.0	1.0
Users	1115	313	2676.11	11.70	0.95 (0.82–1.11)	0.74 (0.59–0.93)*
Fluvastatin						
Nonusers	1106	421	4098.16	10.27	1.0	1.0
Users	1106	386	3791.23	10.18	0.95 (0.83–1.09)	0.84 (0.70–1.02)
Pravastatin						
Nonusers	582	243	2172.62	11.18	1.0	1.0
Users	582	221	2181.54	10.13	0.90 (0.75–1.08)	0.72 (0.56–0.92)*

^*^p < 0.05; **p < 0.001

Covariates adjusted in multivariate models included income, age, sex, area, CHF, CVA, PVD, DM, ASHD, COPD, GI disease, liver disease, dysrhythmia, CABG, PCI, ICD, drugs prescribed before index date (included NSAID, aspirin, acetaminophen, ACEI, ARB, beta-blocker, non-DHP and DHP CCB, acetazolamide, thiazides, loop, potassium sparing diuretics, alpha-blocker, biguanides, sulfonylureas, alpha glucosidase inhibitors, thiazolidinediones, meglitinides, insulin, DPP4 inhibitors, statins, morphine, warfarin, clopidogrel, digoxin).

**Table 5:** Hazard ratio of permanent hemodialysis access recreation for statin users in propensity-score matched cohort.

	N	Event	Person-years	Event Rate (per 100 pearson-years)	Hazard ratio
Overall					
Statin nonusers	3,181	1,028	11908.13	8.63	1.0
Statin users	3,864	1,144	13864.40	8.25	0.94 (0.86–1.01)
AVF					
Statin nonusers	2,736	786	10762.77	7.30	1.0
Statin users	3,267	815	12474.59	6.53	0.88 (0.80–0.97)^*^
AVG					
Statin nonusers	445	242	1145.36	21.13	1.0
Statin users	597	329	1389.81	23.67	1.09 (0.93–1.29)

^*^p < 0.05; **p < 0.001.
