# Supplementary material for: Statins Improve Long Term Patency of Arteriovenous Fistula for Hemodialysis
Source: Sci Rep. 2016 Feb 23;6:22197. doi: 10.1038/srep22197 (PMC4763284; doi:10.1038/srep22197)
Supplement: Supplementary Information [file srep22197-s1.pdf]

Supplementary Information

Statins Improve Long Term Patency of Arteriovenous Fistula for Hemodialysis

Hao-Hsiang Chang MD, MSc[1,2], Yu-Kang Chang PhD[3], Chia-Wen Lu MD[1], Chi-Ting Huang[3], Chiang-Ting Chien PhD[2], Kuan-Yu Hung MD, PhD[4] , Kuo-Chin Huang MD, PhD[1,3,5], Chih-Cheng Hsu, MD, DrPH[1,3,6,7]

[1]Department of Family Medicine, National Taiwan University Hospital, Taipei, Taiwan

[2]Department of Life Science, National Taiwan Normal University, Taipei, Taiwan

[3]Institute of Population Health Sciences, National Health Research Institutes, Zhunan, Miaoli County, Taiwan

[4]Department of Nephrology, National Taiwan University Hospital, Taipei, Taiwan

[5]Department of Family Medicine, College of Medicine, National Taiwan University, Taipei, Taiwan

[6]Department of Health Services Administration, China Medical University, Taichung City, Taiwan.

[7]Institute of Clinical Medicine, National Yang Ming University, Taipei, Taiwan

Correspondence and reprint requests should be addressed to:

Chih-Cheng Hsu, MD, DrPH, Institute of Population Health Sciences, National Health Research Institutes, 35, Keyan Road, Zhunan Town, Miaoli County 350, Taiwan. Tel: 886-37-246166 ext 36336; Fax: 886-37-586-261; E-mail: cch@nhri.org.tw.

or

Kuo-Chin Huang MD, PhD, Department of Family Medicine, National Taiwan University Hospital, Taipei, Taiwan. Address: 7 Chung-Shan South Road, Taipei, Taiwan 100. Tel: 886-2-23123456ext 66081; Fax: 886-2-23118674; E-mail: bretthuang@ntu.edu.tw

Supplementary table S1. Demographic characteristics, comorbid diseases and medications exposure between statin users and nonusers in the propensity-score matched cohort

	Statin nonusers N=3,181		Statin users N=3,864		Standardized Difference
Follow up time (year)					-0.06117
Means (SD)	3.74	(2.61)	3.59	(2.47)	
Age (year)					0.03873
Means (SD)	59.3	(13.1)	59.8	(12.4)	
Sex (n, %)					-0.00033
Male	1279	(40.2)	1553	(40.2)	
Female	1902	(59.8)	2311	(59.8)	
Location (n, %)					0.018828
City	865	(27.2)	1046	(27.1)	
Township	1095	(34.4)	1281	(33.1)	
Rural area	1221	(38.4)	1537	(39.8)	
Comorbidity					
CHF	691	(21.7)	873	(22.6)	0.020957
CVA	502	(15.8)	615	(15.9)	0.003695
PVD	198	(6.2)	264	(6.8)	0.024605
DM	1613	(50.7)	1992	(51.6)	0.016912
ASHD	841	(26.4)	1054	(27.3)	0.018932
Other heart disease	340	(10.7)	440	(11.4)	0.022295
COPD	402	(12.6)	517	(13.4)	0.022067
GI disease	816	(25.7)	1070	(27.7)	0.046116
Liver disease	295	(9.3)	379	(9.8)	0.018198
Cancer	178	(5.6)	244	(6.3)	0.03038
Medication use					
NSAID	2076	(65.3)	2552	(66.1)	0.016488
Aspirin	889	(28.0)	1142	(29.6)	0.035521
Clopidogrel	127	(4.0)	170	(4.4)	0.020304
Warfarin	36	(1.1)	41	(1.1)	-0.00678
ACEI	1207	(37.9)	1497	(38.7)	0.016415
ARB	1186	(37.3)	1477	(38.2)	0.019405
Beta-blocker	1708	(53.7)	2101	(54.4)	0.013641
Non-DHP CCB	468	(14.7)	616	(15.9)	0.034134
DHP CCB	2484	(78.1)	3035	(78.6)	0.011086
Biguanide	516	(16.2)	637	(16.5)	0.007142

Sulfonylurea	929	(29.2)	1122	(29.0)	-0.00368
Alpah glucosidase inhibitor	195	(6.1)	253	(6.6)	0.017131
Thiazolidinedione	159	(5.0)	202	(5.2)	0.010409
Meglitinide	329	(10.3)	435	(11.3)	0.029482
Insulin	836	(26.3)	1080	(28.0)	0.03755
Statin	0	(0.0)	0	(0.0)	-

Abbreviations: CHF, congestive heart failure; CVA, cerebral vascular accident; PVD, peripheral vascular disease; DM, diabetes mellitus; ASHD, atherosclerotic heart disease; COPD, chronic obstructive pulmonary disease; GI, gastrointestinal; NSAID, non steroid anti-inflammatory drug; ACEI, angiotensin converting enzyme inhibitor; ARB, angiotensin II receptor blocker; DHP, dihydropyridine; CCB, calcium channel blocker.

Supplementary table S2. The logistic regression model used for selecting the propensity-score matched cohort

parameter	β	SE	P value	OR	95%CI	
Age (ref=20-39)						
40-49	-0.21	0.08	0.0126	0.81	0.69	0.96
50-59	-0.40	0.08	<.0001	0.67	0.57	0.78
60-69	-0.20	0.08	0.012	0.82	0.70	0.96
≧70	0.12	0.08	0.1558	1.12	0.96	1.32
Male	0.57	0.04	<.0001	1.78	1.65	1.91
Location (ref= City)						
Township	0.02	0.05	0.7159	1.02	0.93	1.11
Rural area	0.23	0.04	<.0001	1.26	1.16	1.37
Income (ref=Middle income)						
poor	0.18	0.16	0.2513	1.20	0.88	1.65
Low income	0.02	0.04	0.6605	1.02	0.93	1.11
High income	0.03	0.07	0.7158	1.03	0.89	1.19
Comorbidity						
CHF	0.05	0.05	0.3302	1.05	0.95	1.15
CVA	-0.07	0.05	0.1502	0.93	0.84	1.03
PVD	0.06	0.07	0.41	1.06	0.93	1.21
DM	-0.39	0.05	<.0001	0.68	0.62	0.75
ASHD	-0.08	0.05	0.0711	0.92	0.84	1.01
Other heart disease	0.09	0.06	0.1108	1.10	0.98	1.22
COPD	0.11	0.05	0.0308	1.12	1.01	1.24
GI disease	0.07	0.04	0.0804	1.07	0.99	1.16
Liver disease	0.37	0.06	<.0001	1.44	1.29	1.62
Cancer	0.01	0.07	0.878	1.01	0.88	1.16
Medication use						
NSAID	0.04	0.04	0.348	1.04	0.96	1.12
Aspirin	-0.19	0.04	<.0001	0.83	0.76	0.90
Clopidogrel	-0.33	0.09	0.0004	0.72	0.60	0.86
Warfarin	0.00	0.17	0.9878	1.00	0.72	1.38
ACEI	0.04	0.04	0.3185	1.04	0.96	1.12
ARB	-0.06	0.04	0.1027	0.94	0.87	1.01
Beta-blocker	-0.16	0.04	<.0001	0.85	0.79	0.92
Non-DHP CCB	0.02	0.05	0.7319	1.02	0.92	1.12
DHP CCB	-0.22	0.05	<.0001	0.80	0.73	0.88
Biguanide	-0.07	0.06	0.2628	0.94	0.84	1.05

Sulfonylurea	-0.08	0.06	0.1491	0.92	0.83	1.03
Alpah glucosidase inhibitor	-0.22	0.08	0.0042	0.80	0.69	0.93
Thiazolidinedione	-0.32	0.09	0.0002	0.73	0.61	0.86
Meglitinide	0.03	0.06	0.6586	1.03	0.91	1.16
Insulin	0.00	0.05	0.9795	1.00	0.91	1.09

Note: the Cox & Snell R-square: 0.4254; the Nagelkerke R-square: 0.6254; the Hosmer and Lemeshow Goodness-of-fit test: P=0.8956

Abbreviations: CHF, congestive heart failure; CVA, cerebral vascular accident; PVD, peripheral vascular disease; DM, diabetes mellitus; ASHD, atherosclerotic heart disease; COPD, chronic obstructive pulmonary disease; GI, gastrointestinal; NSAID, non steroid anti-inflammatory drug; ACEI, angiotensin converting enzyme inhibitor; ARB, angiotensin II receptor blocker; DHP, dihydropyridine; CCB, calcium channel blocker.

Supplementary table S3. Hazard ratio of permanent hemodialysis access recreation for statin users by time-dependent Cox regression models

	HR
Overall	0.80 (0.73-0.88)**
AVF	0.80 (0.72-0.90)**
AVG	0.84 (0.70-1.00)

*p<0.05; **p<0.001

Supplementary table S4. Competing-risk adjusted hazard ratio of permanent hemodialysis access recreation for statin users compared to nonusers

	HR
Overall	0.92 (0.85-1.00)
AVF	0.87 (0.79-0.96)*
AVG	1.07 (0.90-1.25)

*p<0.05; **p<0.001

※ The probability of death and renal transplantation was considered as competing risks adjusted in the model.

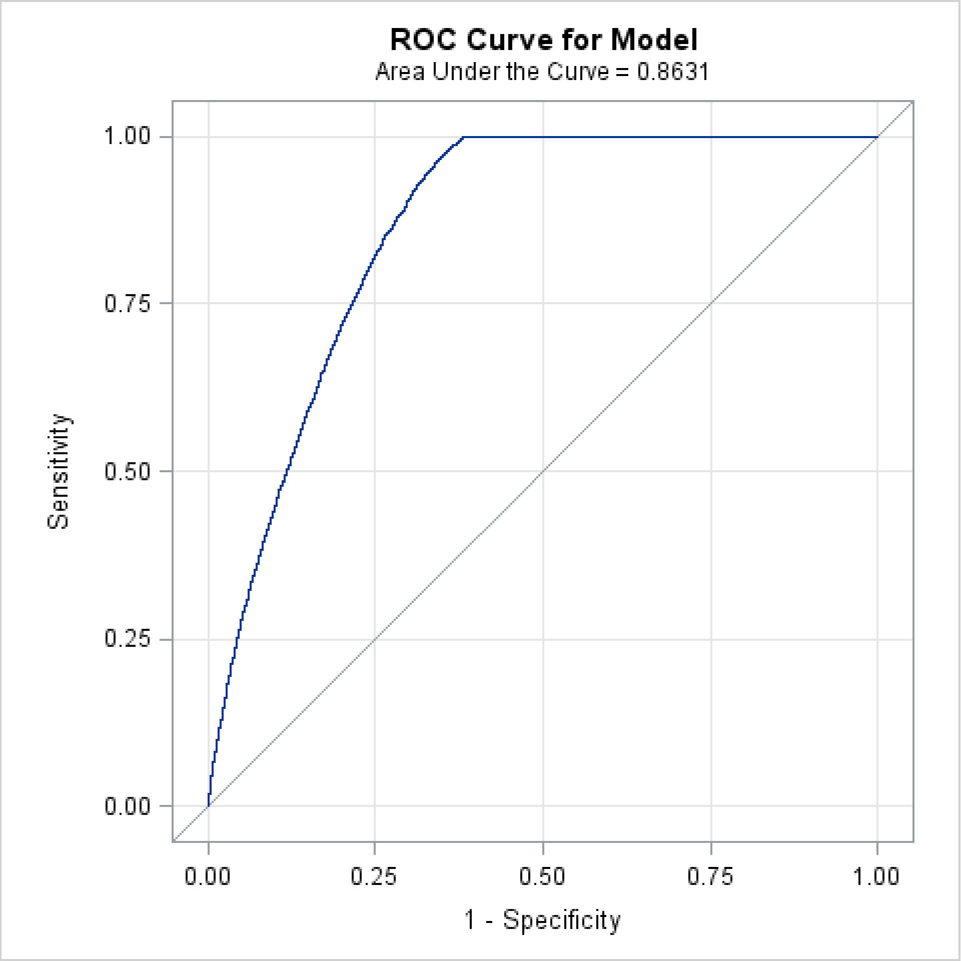

Supplementary figure S1. ROC curve for the logistic regression used for creating propensity scores. The area under curve is o.8631.
